# Contrasting associations between wages and staffing levels of nurses and physicians in Swiss acute care hospitals

**DOI:** 10.3389/frhs.2026.1836914

**Published:** 2026-05-18

**Authors:** Aleksandra Vasic, Michael Simon, Jana Bartakova

**Affiliations:** 1 Department Public Health, Institute of Nursing Science, University of Basel, Basel, Switzerland; 2 St. Claraspital, Medical Clinic, Basel, Switzerland; 3 Department of Public Health, Health Economics Facility, University of Basel, Basel, Switzerland

**Keywords:** hospitals, nurses, physicians, staffing, wages, acute care, workforce, performance

## Abstract

This brief research report presents incremental evidence from the HONEST sub-study, which previously examined the association between nurse and physician wages and 30-day mortality in Swiss acute care hospitals. Building on this work, the present analysis examines the association between wages and staffing levels, as staffing may represent a potential mechanism linking wage structures to hospital performance. Routinely collected hospital data from the Swiss Federal Office of Statistics covering the years 2018–2020 were analyzed, with 2019 serving as the index year. Staffing levels were calculated as hours per inpatient day based on full-time equivalents and total inpatient days. Associations between wages and staffing were assessed using Spearman's rank correlation coefficients, with analyses stratified by hospital level. Nurse staffing levels were positively correlated with nurse wages across all years, although the associations were modest overall (*ρ* = 0.22–0.38, *p* < 0.001). In contrast, physician staffing levels showed a moderate to strong inverse correlation with wages (*ρ* = −0.56 to −0.61, *p* < 0.001). While the physician results are consistent with typical hospital workforce structures, the positive association between nurse staffing and wages was less expected. These findings highlight the limited empirical evidence on wage–staffing relationships in hospitals and highlight the complexity of workforce dynamics. Given the descriptive and cross-sectional design, the results should not be interpreted as causal, and the underlying mechanisms remain unclear. Further research is needed to better understand how wage structures and workforce composition are associated with staffing patterns in healthcare systems.

## 1 Introduction

This brief research report presents incremental evidence from the HONEST sub-study, which examined the association between nurse and physician wages and 30-day mortality as a patient safety outcome in Swiss acute care hospitals (1). Using the same routinely collected data which was provided from the Swiss Federal Office of Statistics (FSO), the present analysis focuses exclusively on hospital-level information to further elucidate the relationship between wage structures and staffing-relevant outcomes in the Swiss acute care setting.

In the HONEST sub-study, no association was observed between nurse wages and 30-day mortality, whereas a weak but statistically significant association was observed for physician wages, with higher wages being associated with a 2% reduction in mortality risk [OR: 0.98 (95% CI: 0.96–0.99), *p* = 0.001]. These findings should not be interpreted as causal and must be considered within the broader health services and workforce policy context, where hospital ownership, organizational characteristics, job satisfaction, and other institutional factors may influence both wage structures and patient outcomes (1–4).

Staffing levels represent one potential mechanism within this context. Nurse staffing is a well-established determinant of hospital quality and patient safety, with longitudinal evidence linking higher registered nurse staffing to lower mortality (5, 6). Emerging evidence also suggests that physician staffing levels may be associated with hospital outcomes (7, 8). Wage structures may influence staffing indirectly by shaping labor supply and affecting recruitment and retention (9, 10). However, empirical research directly examining wage–staffing relationships within hospitals remains limited (11, 12).

Consequently, it remains unclear whether higher wage levels under constrained financial resources are associated with lower staffing levels—potentially accompanied by a more highly qualified workforce—or whether lower wages that enable higher staffing levels may be more favorable for care delivery. To explore this potential trade-off, the present analysis examines the association between wages and staffing levels in Swiss acute care hospitals.

## 2 Methods

### 2.1 Design, setting and sample

This secondary analysis used routinely collected Swiss hospital data from 2018 to 2020 provided by the FSO under a signed data protection contract in accordance with Article 22 of the Swiss Federal Act on Data Protection (13). The main analysis used 2019 as the index year to minimize potential confounding related to the COVID-19 pandemic, while data from 2018 to 2020 were used for sensitivity analyses.

The study focused on general acute care hospitals in Switzerland. Hospitals are classified by the FSO into five levels based on annual patient volume and their role in clinical education (14), with Level 1 representing the highest level of accreditation. Levels 1–4 were included, corresponding to university hospitals, tertiary care hospitals (cantonal) and large and medium primary care hospitals. Level 5 hospitals, which primarily comprise small regional hospitals as well as specialty clinics [rehabilitation, mental health, pediatric, gynaecological/neonatal, geriatric and special surgical clinics as defined by the FSO (14)], were excluded due to differing care profiles and patient outcomes, in order to reduce institutional heterogeneity.

The sample consisted of nurses and physicians. Nursing staff comprised registered nurses with a tertiary diploma or bachelor's degree (including midwives), licensed practical nurses (“Fachperson Gesundheit”), and nursing assistants. Medical staff included physicians with a medical diploma and junior physicians (“Unterassistenzärzt*innen”). Other healthcare professionals and non-clinical staff were excluded.

### 2.2 Data source

The data originates from a mandatory nationwide hospital survey conducted by the FSO, which collects standardized hospital statistics from all Swiss hospitals (15). The hospital statistics dataset comprises five sub-datasets: general information, services, personnel, costs and wages (16).

### 2.3 Variables and measurements

For this secondary analysis we used the variables *net wages* and *staffing*. *Net wages* represent remuneration after deduction of compulsory social insurance contributions and are reported in Swiss Francs (CHF) per full-time equivalent (FTE). Wage data were obtained from the mandatory FSO hospital survey and are available at the institutional level as aggregated annual wage expenditures for employee groups, including nurses and physicians.

Staffing information, including the number of employees and their working time in FTE, was derived from the same data source and aggregated annually per employee group and hospital to ensure consistency with the wage measure. The *staffing* variable reflects realized workforce allocation at the hospital level and is therefore interpreted as a measure of labor demand rather than underlying labor supply.

The *staffing* variable was calculated following the approach described by Simon et al. (17). For nursing staff, the FTE were converted into hours per inpatient nursing day by multiplying the FTE by 1,890 (based on a 42-h workweek and an average of 45 working weeks per year). These annual hours were then divided by the total number of inpatient days (variable “*Pflegetage_stationaer*”), which represents the total number of patient days provided by the hospital within a year. The resulting measure reflects the average number of nursing staff hours available per inpatient day.

The same approach was applied for physicians, using 2,475 h per FTE (based on a 55 h workweek) (18). Staffing levels are therefore expressed as hours per inpatient day and should not be interpreted as percentages. For example, a value of 10 indicates that, on average, 10 h of staff time are available per inpatient day.

### 2.4 Data access and cleaning

The data were accessible based on a signed data protection agreement with the FSO. Data cleaning, merging and aggregation followed the methodology established in the predecessor study to the HONEST sub-study by Holzer et al. Specifically, we applied the same extensive bounds for net wages ranging from 30,000 to 200,000 CHF per FTE per year for nurses and 60,000–400,000 CHF per FTE per year for physicians (19).

### 2.5 Data analysis

The unit of analysis is the hospital, with all variables aggregated at the hospital level. The analysis is purely descriptive and focuses on bivariate associations between wages and staffing levels.

Spearman's rank correlation coefficients (*ρ*) were calculated between *net wages* and the corresponding *staffing* variable. Analyses were additionally stratified by hospital level to partially account for structural differences in case mix and organizational complexity across hospital types. Spearman's method was chosen to account for potential non-linear relationships and non-normal distributions of the variables. As rank correlations are invariant to monotonic transformations, wages were not log-transformed, and no inflation adjustment was applied, as analyses were conducted separately for each year.

No regression models were estimated or interpreted as part of the analysis, and the study does not aim to estimate directional or causal effects. For visualization, scatterplots with fitted linear regression lines were generated separately for nurses and physicians each, with data points stratified by hospital level and regression lines shown for each level as well as for the overall sample. These regression lines are included for visualization purposes only, to illustrate the association between wages and staffing variables.

Sensitivity analyses were conducted using data from 2018 to 2020, applying the same analytical procedures as for 2019. The sample comprised 81 hospitals in 2018, 82 in 2019, and 83 in 2020. All analyses were performed using the statistical software “R” (version 4.3.3; 2024-02-29) (20), with the packages “dplyr” (21), “ggplot2” (22) and “scales” (23).

## 3 Results

The following results describe bivariate associations between wages and staffing levels at the hospital level, based on Spearman's rank correlation coefficients. Descriptive statistics and correlation results for the index year (2019) are presented in Tables 1, 2. Corresponding results for the sensitivity analyses (2018 and 2020) are provided in the Supplementary Tables S1–S4.

**Table 1:** Descriptive statistics, 2019.

Variable	Minimum	Maximum	Mean (SD)
Nurses net wages (CHF/FTE/year)	45,251.7	124,229.8	80,318.5 (±16,287.4)
Physician net wages (CHF/FTE/year)	111,318.9	393,634.8	208,007.7 (±51,644.5)
Nurse staffing (h/inpatient day)	9.2	29.5	14.8 (±3.7)
Physician staffing (h/inpatient day)	0.5	15.0	6.7 (±3.2)

SD, standard deviation; CHF, Swiss francs; FTE, full time equivalent; h, hours.

**Table 2:** Spearman's rank correlation coefficient (*ρ*) between staffing levels and net wages for nurses and physicians, 2019.

Variable	Overall *ρ*	*ρ* stratified by hospital level				*p*
		Level 1	Level 2	Level 3	Level 4	
Nurses	0.38	0.90	0.05	0.34	0.31	<0.001
Physicians	−0.61	−0.26	−0.45	−0.04	−0.28	<0.001

*Ρ*, Spearman's rank correlation coefficient.

### 3.1 Correlation results

Nurse staffing levels were positively correlated with net wages (*ρ* = 0.38, *p* < 0.001), indicating a positive association at the hospital level. Stratified analyses showed substantial variation across hospital levels, with the strongest correlation observed in Level 1 hospitals (*ρ* = 0.90, *p* < 0.001) and a weak association in Level 2 hospitals (*ρ* = 0.05, *p* < 0.001).

In contrast, physician staffing levels were negatively correlated with net wages (*ρ* = −0.61, *p* < 0.001), indicating an inverse association at the hospital level. Stratified analyses showed negative associations across all hospital levels, with the strongest correlation in Level 2 hospitals (*ρ* = −0.45, *p* < 0.001) and the weakest in Level 3 hospitals (*ρ* = −0.04, *p* < 0.001).

### 3.2 Graphical representation

**Figure 1:**
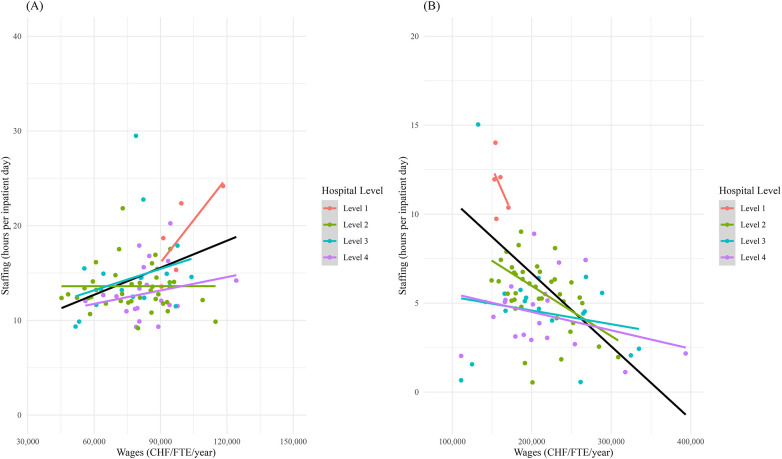
Scatterplot of wages and staffing levels, 2019. **(A)** nurses, **(B)** physicians.

Figure 1 illustrates the association between wages and staffing variables. Scatterplots with fitted linear regression lines are presented for visualization purposes only, including both hospital-level-specific and overall trends.

### 3.3 Sensitivity analyses

Sensitivity analyses for 2018 and 2020 yielded results comparable to the main analysis. For nurses, correlations remained positive in both years (*ρ* = 0.30, *p* < 0.001 in 2018; *ρ* = 0.22, *p* < 0.001 in 2020), with the strongest associations observed in Level 1 hospitals and weak or negative associations in Level 2 hospitals.

For physicians, correlations remained consistently negative (*ρ* = −0.59, *p* < 0.001 in 2018; *ρ* = −0.56, *p* < 0.001 in 2020), with the strongest associations in Level 2 hospitals and weaker associations in Level 3 hospitals.

Overall, the patterns of association were consistent across years with minor differences in Level 3 regression slopes for nurses. Corresponding tables and scatterplots for 2018 and 2020 are provided in the Supplementary Figures S1, S2.

## 4 Discussion

Our analysis reflects realized staffing levels at the hospital level and is therefore best interpreted from a demand-side perspective. While wage structures may influence labor supply through recruitment and retention mechanisms, the broader labor supply is not captured in our data. Previous research has distinguished between labor demand and supply by combining FTE-based staffing measures with headcount data, for example in the UK context where headcount can serve as a proxy for available workforce supply (24). In contrast to more centralized systems such as the UK, where workforce headcount may approximate the available labor supply due to the dominant role of a single public employer, hospital-based data in Switzerland capture only employed staff and therefore primarily reflect realized labor demand. This is partly due to the more decentralized structure of the Swiss healthcare system, where available data do not include comprehensive measures of the potential workforce across the system (25). Also, the observed associations should not be interpreted as causal relationships. While wage levels may influence staffing through recruitment and retention mechanisms, reverse causality is also plausible, as staffing levels and workforce composition may affect average wages. Given the cross-sectional and correlational design, the direction of these relationships cannot be determined.

Nurse staffing levels were positively but modestly associated with wages across all years, with substantial variation across hospital levels. The relationship ranged from very strong correlations in Level 1 hospitals (*ρ* ≈ 0.90) to minimal or near-zero correlations in other hospital levels (e.g., Level 2 hospitals), despite statistical significance. This pattern was consistent across the sensitivity analyses for 2018 and 2020, suggesting that these extreme values are not driven by a single year. While the associations, particularly among nurses, are statistically significant, their magnitude is modest. Statistical significance reflects the likelihood that an observed association is not due to chance, whereas practical or policy relevance depends on the strength and implications of the relationship. Therefore, these findings should be interpreted with caution, as their practical significance may be limited and the observed associations show considerable heterogeneity across hospital levels.

From a conventional economic perspective, a positive association between nurse staffing levels and wages may appear counterintuitive, as hospitals might be expected to face budget constraints that lead to trade-offs between staffing levels and wage expenditures. However, our findings (especially the strong association in Level 1 hospitals) suggest that this simplified trade-off model may not hold universally. An alternative interpretation of the contrasting patterns observed for nurses and physicians can be framed using standard labor market theory (26, 27). The inverse association between physician wages and staffing may reflect short-run movement along a downward-sloping labor demand curve, where hospitals facing higher wages adjust by employing fewer physicians, particularly in a context of relatively constrained short-term labor supply. In contrast, the positive association observed for nurses may be indicative not only of movement along the demand curve but also of outward shifts in labor demand, potentially driven by increasing care needs, policy attention, or institutional priorities to strengthen nursing capacity. If accompanied by a more elastic labor supply–through mechanisms such as part-time work, re-entry into the workforce, or expanded training capacity–such demand shifts could result in simultaneous increases in both wages and staffing levels. Although our data do not allow us to distinguish between these mechanisms, this framework provides a plausible explanation for the differing patterns observed between professional groups. A further explanation is that level 1 hospitals handle more complex cases with higher patient acuity, which may be associated with both greater nurse-to-patient ratios and a more specialized, highly qualified nursing workforce (28–30). In such settings, hospitals may require both larger nursing teams and competitive wages to attract and retain staff with the required expertise.

Temporal patterns suggest that external factors may also have influenced staffing and wage dynamics. The decline in correlation from 2019 to 2020 may reflect disruptions associated with the COVID-19 pandemic, during which increased demand for healthcare personnel was, in some settings, addressed through alternative staffing arrangements such as the involvement of students, retired professionals, or volunteers (31, 32), potentially also affecting wage structures. However, these considerations are largely based on general observations from pandemic responses or international studies. To our knowledge, such effects have not been systematically examined in the Swiss acute care context.

From a policy perspective, these findings are timely given recent developments in Switzerland's healthcare system. In 2021, Swiss voters approved a “Nursing Initiative” that obliges the federal government and cantons to enact measures to strengthen the nursing profession (33, 34). These measures aim to ensure safe staffing levels and improve working conditions, primarily by increasing the number of trained nurses and enhancing job quality. tBecause staffing levels and compensation are central targets of this initiative, monitoring trends in nurse staffing and wages in the coming years will be particularly important to evaluate the impact of the reforms. However, further research is needed to assess whether such policy measures lead to causal changes in staffing–wage relationships.

In contrast to nurses, physician staffing levels were negatively associated with wages. Hospitals with higher physician staffing tended to report lower average physician wages. This inverse association was moderate to strong overall and most pronounced in Level 2 hospitals. This pattern is consistent with typical workforce structures, where hospitals with larger physician workforces may employ a higher proportion of residents and junior physicians, who receive lower salaries compared to senior physicians (35). Conversely, smaller hospitals tend to have fewer residents and relatively more senior doctors. Thus, facilities with high physician staffing often have a lower average wage (due to the prevalence of residents and junior physicians), whereas those with lower staffing levels have a higher average wage. At the same time, some of the observed correlations were close to zero despite statistical significance (e.g., Level 3 hospitals in 2019), indicating that these results should also be interpreted in light of their magnitude and the underlying workforce composition.

Ongoing workforce policies and debates may further influence physician staffing and compensation. In Switzerland, there has been active discussion about capping physicians' working hours. Some reports cite an average physician work week of around 50 h (18), whereas others suggest that 56 h or more per week are common. Such long hours have raised concerns about patient care quality and physician well-being, leading the Union of Swiss Assistant and Senior Doctors (Verband Schweizerischer Assistenz- und Oberärztinnen und -ärzte) to advocate for a 42 h standard work week, with an additional four hours allocated for training (36). Should regulations emerge to limit working hours, hospitals might need to hire additional physicians or redistribute workloads, which could alter staffing levels, skill mix, and wage structures. Future studies will be needed to observe how such policy changes might affect the staffing–wage relationship among physicians.

Our analysis also highlighted a marked difference in wage ranges between the nursing and physician workforces. This disparity is possibly attributable in part to the differences in education and training: becoming a physician generally requires a longer and more specialized training pathway, which commands higher compensation. It remains unclear, however, whether these factors alone fully explain the wage gap between the professions. Other influences—such as the gender composition differences between nursing and medical staff and any associated gender pay gap effects, as suggested by Holzer et al. (19)—may also contribute to the observed wage disparity. A detailed investigation of these aspects was beyond the scope of our study, but it warrants attention in future research.

This study has several limitations that warrant consideration. First, it relies on aggregated hospital-level data, which limits the ability to capture within-hospital variability and detailed workforce composition. The analysis reflects average patterns and may mask important heterogeneity across departments, units, or staff roles. Consequently, the findings should be interpreted as hospital-level associations, as individual wage distributions and specific staff mix within hospitals could not be examined, and may therefore not directly generalize to individual- or unit-level dynamics. More granular data would be required to investigate these aspects in greater detail. In addition, the relatively small number of Level 1 hospitals further limits comparability across hospital categories.

Second, the analysis is descriptive and does not fully account for potentially relevant factors such as detailed hospital characteristics, staff skill mix, working conditions, organizational environment, regional economic conditions, or patient case mix, all of which may influence staffing and wage patterns. Although the analyses were stratified by hospital level to partially account for structural differences (e.g., case mix, organizational complexity, and skill mix), this approach does not substitute for direct measurement or multivariable adjustment, and unmeasured factors may still influence the observed associations. Skill-mix distribution by hospital level has been reported in a previous publication by Holzer et al. (19), but could not be directly included in the presented analysis, as only aggregated wage data were available.

In particular, hospital-level heterogeneity–such as differences in working conditions, organizational environment, and job satisfaction–may affect workforce dynamics through recruitment and retention mechanisms but is not captured in the aggregated data. While the case mix index is primarily designed for hospital reimbursement and is not a direct measure of patient acuity (37), it could be used as a proxy for hospital case complexity in future studies. Third, the cross-sectional design precludes causal inference, and reverse causality cannot be ruled out. The observed relationships should therefore be interpreted as associations rather than causal effects.

Future research using more granular and longitudinal data (extending beyond 2020), as well as multivariable modeling approaches, could help validate and extend these findings. Despite these limitations, this study provides an initial perspective on the interplay between staffing levels and wages for nurses and physicians and offers a foundation for further investigation.

## Data Availability

The datasets presented in this article are not readily available because the data, including derived datasets, are subject to a confidentiality agreement with the Swiss Federal Office of Statistics. The statistical code (excluding any data-related content) is publicly available on GitHub, an open-access repository. Requests to access the datasets should be directed to https://www.bfs.admin.ch/bfs/en/home/services/contact.html.

## Supplementary material

Supplementary Table S1 Descriptive statistics, 2018

Supplementary Table S2 Descriptive statistics, 2020

Supplementary Table S3 Spearman's rank correlation coefficient (ρ) between staffing levels and net wages for nurses and physicians, 2018

Supplementary Table S4 Spearman's rank correlation coefficient (ρ) between staffing levels and net wages for nurses and physicians, 2020

Supplementary Figure S1 Scatterplot of Wages and Staffing Levels, 2018. **(A)** Nurses, **(B)** Physicians.

Supplementary Figure S2 Scatterplot of Wages and Staffing Levels, 2020. **(A)** Nurses, **(B)** Physicians.
