# Supplementary material for: Contrasting associations between wages and staffing levels of nurses and physicians in Swiss acute care hospitals
Source: Front Health Serv. 2026 May 18;6:1836914. doi: 10.3389/frhs.2026.1836914 (PMC13222961; doi:10.3389/frhs.2026.1836914)
Supplement: Supplementary Table S1 — Descriptive statistics, 2018 [file Table1.docx]

Table S1 Descriptive statistics, 2018

| **Variable** | **Minimum** | **Maximum** | **Mean (SD)** |
| --- | --- | --- | --- |
| Nurses Net Wages (CHF/FTE/year) | 37,572.7 | 140,644.6 | 79,451.5 (±18,592.9) |
| Physician Net Wages (CHF/FTE/year) | 120,112.8 | 379,095.9 | 207,148.4 (±52,587.7) |
| Nurse Staffing (h/inpatient day) | 8.8 | 25.3 | 14.5 (±3.9) |
| Physician Staffing (h/inpatient day) | 0.5 | 13.8 | 6.5 (±3.1) |

SD: standard deviation; CHF: Swiss francs; FTE: full time equivalent; h: hours
