# Supplementary material for: Contrasting associations between wages and staffing levels of nurses and physicians in Swiss acute care hospitals
Source: Front Health Serv. 2026 May 18;6:1836914. doi: 10.3389/frhs.2026.1836914 (PMC13222961; doi:10.3389/frhs.2026.1836914)
Supplement: Supplementary Table S2 — Descriptive statistics, 2020 [file Table2.docx]

Table S2 Descriptive statistics, 2020

| **Variable** | **Minimum** | **Maximum** | **Mean (SD)** |
| --- | --- | --- | --- |
| Nurses Net Wages (CHF/FTE/year) | 44,535.9 | 115,061.6 | 80,437.4 (±15,707.2) |
| Physician Net Wages (CHF/FTE/year) | 81,506.3 | 356,850.5 | 211,452.6 (±51,511.9) |
| Nurse Staffing (h/inpatient day) | 9.5 | 37.2 | 16.3 (±4.5) |
| Physician Staffing (h/inpatient day) | 0.6 | 18.2 | 7.5 (±3.7) |

SD: standard deviation; CHF: Swiss francs; FTE: full time equivalent; h: hours
