# Supplementary material for: Contrasting associations between wages and staffing levels of nurses and physicians in Swiss acute care hospitals
Source: Front Health Serv. 2026 May 18;6:1836914. doi: 10.3389/frhs.2026.1836914 (PMC13222961; doi:10.3389/frhs.2026.1836914)
Supplement: Supplementary Table S3 — Spearman's rank correlation coefficient (ρ) between staffing levels and net wages for nurses and physicians, 2018 [file Table3.docx]

Table S3 Spearman’s rank correlation coefficient (ρ) between staffing levels and net wages for nurses and physicians, 2018

| **Variable** | **Overall ρ** | **ρ stratified by hospital level** | | | | **p** |
| --- | --- | --- | --- | --- | --- | --- |
|  |  | Level 1 | Level 2 | Level 3 | Level 4 |  |
| Nurses | 0.30 | 0.90 | -0.05 | 0.05 | 0.48 | < 0.001 |
| Physicians | -0.59 | -0.27 | -0.34 | -0.17 | -0.18 | < 0.001 |

ρ: Spearman’s rank correlation coefficient.
