# Supplementary material for: Contrasting associations between wages and staffing levels of nurses and physicians in Swiss acute care hospitals
Source: Front Health Serv. 2026 May 18;6:1836914. doi: 10.3389/frhs.2026.1836914 (PMC13222961; doi:10.3389/frhs.2026.1836914)
Supplement: Supplementary Table S4 — Spearman's rank correlation coefficient (ρ) between staffing levels and net wages for nurses and physicians, 2020 [file Table4.docx]

Table S4 Spearman’s rank correlation coefficient (ρ) between staffing levels and net wages for nurses and physicians, 2020

| **Variable** | **Overall ρ** | **ρ stratified by hospital level** | | | | **p** |
| --- | --- | --- | --- | --- | --- | --- |
|  |  | Level 1 | Level 2 | Level 3 | Level 4 |  |
| Nurses | 0.22 | 0.91 | -0.17 | 0.20 | 0.42 | < 0.001 |
| Physicians | -0.56 | -0.26 | -0.36 | -0.17 | -0.28 | < 0.001 |

ρ: Spearman’s rank correlation coefficient.
