# Supplementary figures and images for: Contrasting associations between wages and staffing levels of nurses and physicians in Swiss acute care hospitals
Source: Front Health Serv. 2026 May 18;6:1836914. doi: 10.3389/frhs.2026.1836914 (PMC13222961; doi:10.3389/frhs.2026.1836914)

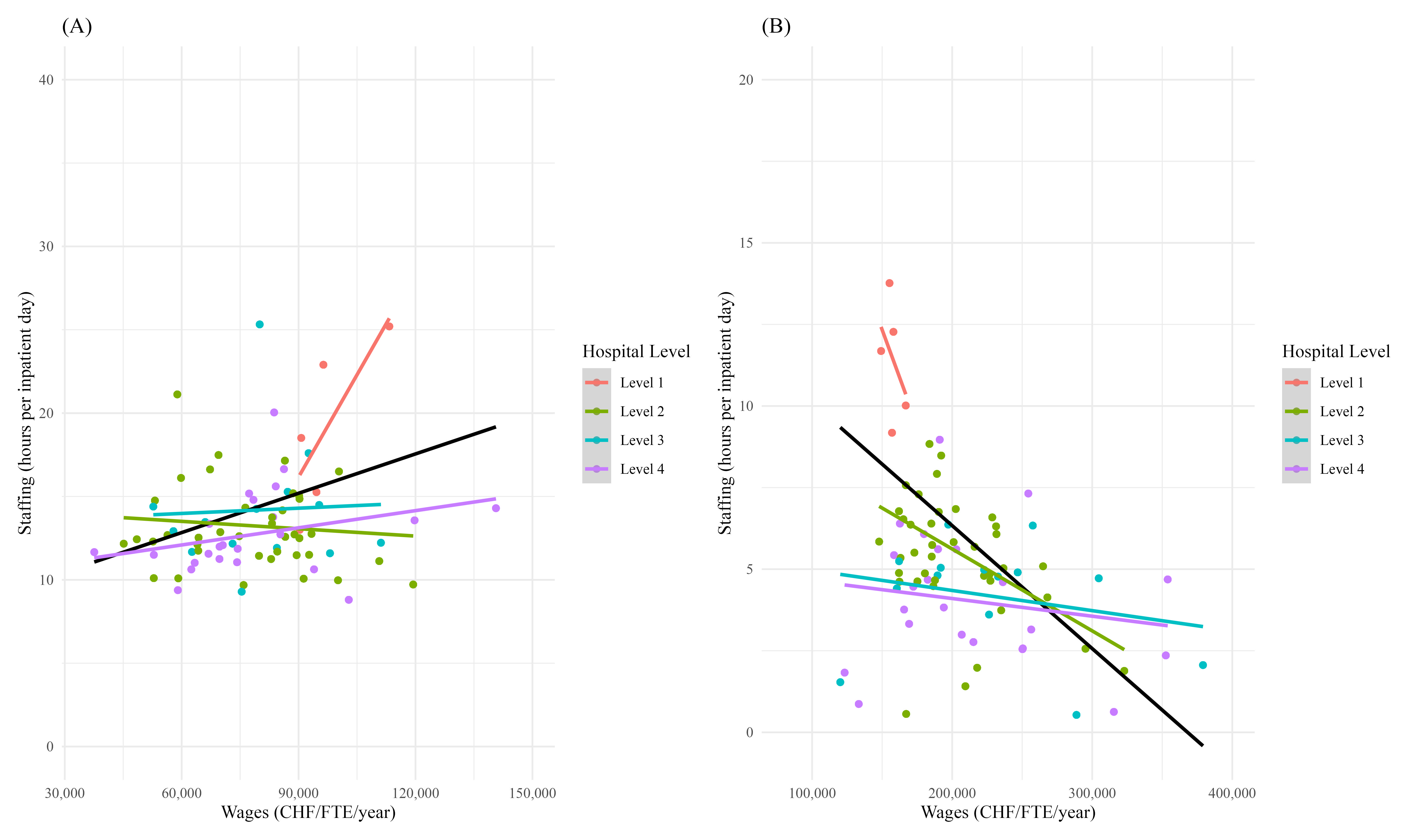

Supplement: Supplementary Figure S1 — Scatterplot of Wages and Staffing Levels, 2018. (A) Nurses, (B) Physicians. [file Image1.jpeg]

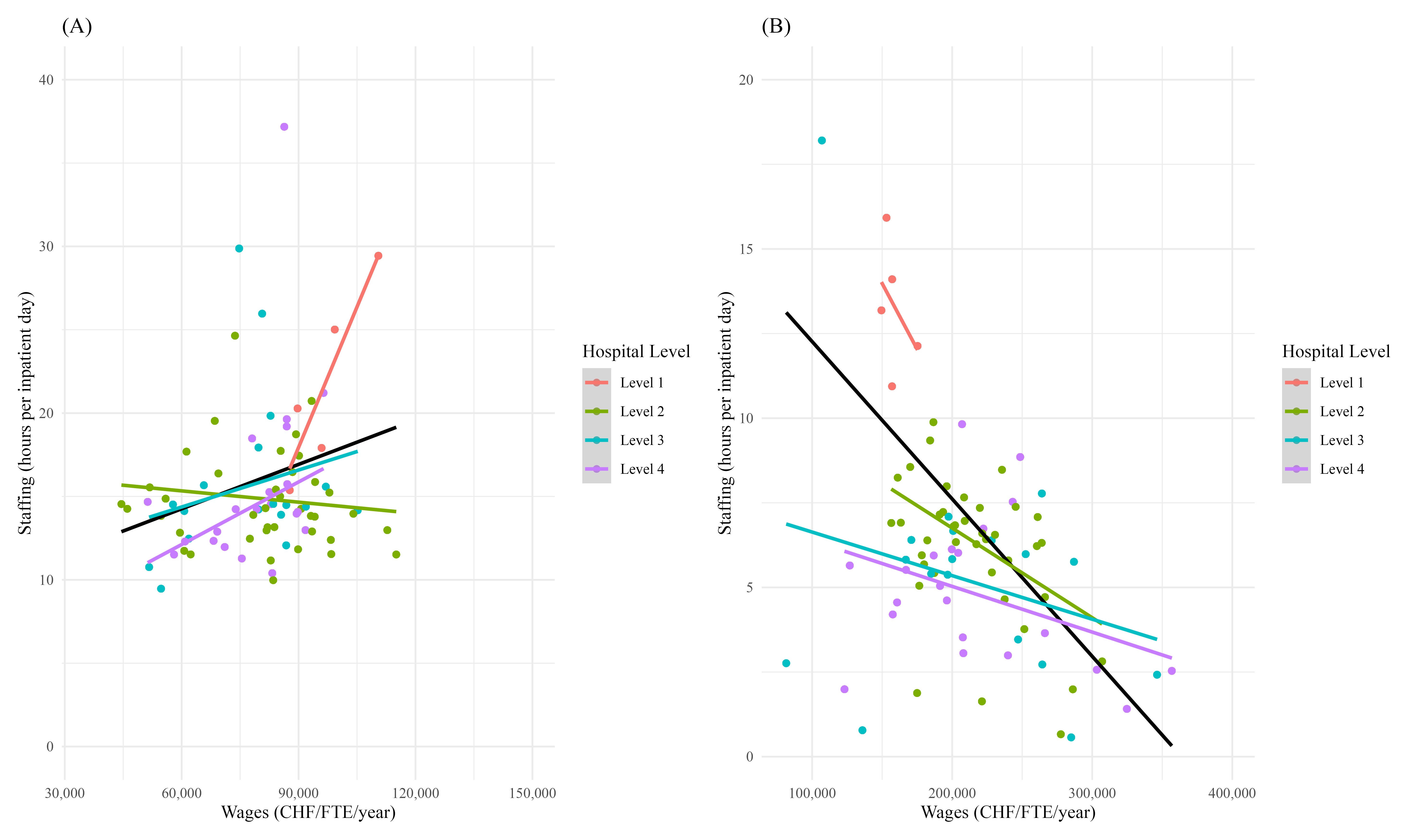

Supplement: Supplementary Figure S2 — Scatterplot of Wages and Staffing Levels, 2020. (A) Nurses, (B) Physicians. [file Image2.jpeg]
